# Impact of Green Supply Chain Management on Sustainable Performance: A Dual Mediated-moderated Analysis of Green Technology Innovation And Big Data Analytics Capability Powered by Artificial Intelligence

**DOI:** 10.12688/f1000research.154615.2

**Published:** 2025-09-08

**Authors:** Quswah Makhdoom, Ikramuddin Junejo, Jan Muhammad Sohu, Syed Mir Muhammad Shah, Belal Mahmoud Alwadi, Faisal Ejaz, Md Billal Hossain

**Affiliations:** 1 Department of Management Sciences, SZABIST University, Hyderabd Campus, Pakistan; 2 School of Management, Jiangsu University, Zhenjiang, Jiangsu, China; 3 Department of Business Administration, Sukkur IBA University, Sukkur, Pakistan; 4 Department of Basic Sciences (Humanities and Scientific), Al-Zaytoonah University of Jordan, Amman, Jordan; 5 School of International Relations, Minhaj University Lahore, Lahore, Punjab, Pakistan; 6 Department of Political Science, University of Okara, Punjab, Pakistan; 7 Sustainability Competence Centre, Faculty of Business and Economics, Szechenyi Istvan Egyetem, Győr, Gyor-Moson-Sopron, Hungary

**Keywords:** Sustainable Performance; Green supply chain Management; Waste management, Green Technology innovation; SMEs; Developing country

## Abstract

**Background:**

This study aims to empirically test a comprehensive interrelationship between green supply chain management (GSCM), green technology innovation (GTI), waste management (WM), big data analytics capability powered by artificial intelligence (BDAC-AI), and their collective impact on sustainable performance (SP) in organizational contexts.

**Methods:**

This study was conducted in Pakistan’s food processing sector. The respondents included 495 managers working in the food processing industry. A structural equation modelling (SEM) approach is used to examine direct and indirect relationships between the variables. The originality of this study lies in integration of the technology acceptance model (TAM) and dynamic capability theory (DCT) to understand sustainable practices in the context of the provided model.

**Results:**

This study highlights that GSCM, GTI, WM, and BDAC-AI have positive, strong, and direct impacts on SP. Furthermore, GTI and WM only partially mediate the link between GSCM and SP, whereas the two moderate the link. In addition, BDAC-AI had a moderating effect on the relationship between GTI and SP. This study has managerial implications, including strategies that involve the use of theoretical frameworks for technological acceptance and dynamic capabilities to support sustainable initiatives. However, it is worth noting that the findings provide a practical contingency for managers and businesses interested in implementing green studies effectively, improving technologies, and strengthening sustainable performance capabilities.

**Conclusions:**

The study extends the literature by establishing a model for operationalizing GSCM in the food processing sector. Furthermore, it adds value in that it first integrates TAM and DCT to explain sustainable operations and their impact on organizations. Furthermore, it extends the existing literature by establishing a relationship between GSCM and SC. It offers a model through which GSCM can be operationalized in the context of the FS sector.

## Introduction

The food sector has become increasingly important in the present era, with short environmental concerns and a growing awareness of problems related to sustainable consumption and production. Global demand places immense pressure on supply chains ( Attinasi et al., 2022), whereas waste management systems incur significant costs ( Kumar et al., 2021). This situation is constantly tight ( Attinasi et al., 2022). However, some critical large-scale limitations limit the full implementation of green supply chain management (GSCM) as a viable concept for sustainable challenges within supply chains ( Debnath et al., 2023). Different organizations are inclined to focus on short-term financial revenue, which may be an environmental perspective, which in turn leads to the issue of limited continuation of GSCM measures in different fields and areas ( Söderholm, 2020). The above knowledge and resources gap is highly original in less developed countries, where GSCM program implementation is often poor ( Rambaldi, 2022).

Neglecting proper waste disposal severely harms the environment and inhabitants’ quality of life internationally ( Kumar et al., 2021). Despite some improvements, there are many difficulties and inequalities in the global community ( Bayu et al., 2022; Yousefloo & Babazadeh, 2020). Some of the issues that can be highlighted are as follows: recycling processes are said to be very slow in this regard ( Bruhn et al., 2023). Most plastic waste does not effectively undergo recycling; however, it ends up in landfills or pollutes ecosystems ( Praveenkumar et al., 2024). The World Bank Report 2022 “Solid Waste Management” indicates that, in contrast to developed countries, recycling rates in low-income countries remain significantly lower; in other words, people generate more trash, and the environment inevitably suffers. However, the state of dealing with and discharging similar wastes differs widely between Asian countries ( Darko et al., 2023). Insufficient waste collection and disposal practices present challenges, such as open dumping and incineration, in several urban areas ( Mor & Ravindra, 2023). According to the World Bank (2020), prioritizing waste management is crucial for Pakistan to ensure the sustainability of its supply chain ( Haque et al., 2023; Ragasri and Sabumon, 2023). The issue of unreliable supply chains is a significant challenge for Pakistan’s food-producing sector in Pakistan ( Tsai et al., 2021). Distribution networks are characterized by the presence of waste, inefficiency, and lack of environmental concern ( Zuberi & Ali, 2015). This chronic issue not only escalates production expenses but also significantly diminishes operational effectiveness, amplifies resource use, and exacerbates environmental consequences ( Papamichael et al., 2024). The future economic prosperity of Pakistan will be greatly impacted by the degree to which the food industry welcomes and incorporates green technology innovation (GTI) in a timely fashion, as stated in the World Bank report in a press release on April 3, 2023. While the environmental and economic benefits of GTI have been extensively discussed, its use in Pakistan’s food industry has not been thoroughly explored ( Yu et al., 2023). The food industry in Pakistan plays a vital role in the country’s economic development and overall well-being of its population ( Ashraf et al., 2023a). The implementation of the GTI presents an opportunity for the food industry in Pakistan to effectively mitigate waste generation, optimize resource utilization, and embrace ecologically sustainable practices ( Rahman et al., 2023). The insufficiency of resources and energy in Pakistan contributes to shortages, inefficiencies, and wastage within the agricultural sector ( Ashley, 2016; Rahman et al., 2023).

Applying big data analytics capability powered by artificial intelligence (BDAC-AI) is the only possible way to significantly increase the food production sectors in Pakistan to a significantly higher level ( Ali et al., 2023). It is possible to gain enhanced decision-making with data fabric technologies to improve industrial processes ( Niu et al., 2023), supply chains ( AL-Khatib, 2022), and product quality ( Saini et al., 2023a) among other areas and provide substantial input to advancing sustainable resource management ( Asha et al., 2023), waste confinement. Pakistan has two main issues: continuing population growth and minimizing environmental impacts ( Waseem & Rana, 2023). Therefore, the available literature indicates that sustainable practices implemented in the food industry improve resource efficiency and emission reduction ( Ashley, 2016), as well as the sustainability of the food value chain ( Asha et al., 2023).

Several studies have theoretically analyzed BDAC, AI, GTI, and GSCM; however, more research is needed to address the food industry ( Feng et al., 2022). This study examined the relationship between GSCM, waste management (WM), GTI, and BDAC-AI in Pakistan’s food industry. These elements comprise the framework of the food industry’s sustainable performance initiatives. The integration of these components offers a favorable background for studying the existence of these factors in sustainable food industry ( Roy et al., 2023). As such, the choice of this model for review stems from its ability to improve the theorization of sustainability and offer prescriptions on how corporations, governments, and individuals can support the movement for a sustainable food system in a developing country.

On several occasions, it has been realized that the food business contributes significantly to employment generation and stimulation of the economy within emerging nations ( Nikseresht et al., 2023). The release of greenhouse gases and the notion of resource depletion are some of the main ways through which this specific behavior contributes to a host of environmental losses ( Bhattacharya & Kalakbandi, 2022; Torres, 2018). Thus, for the continuous stability and profitable future of the food business in the globalized world, which is going to experience enhanced ecological volatilities, the sector must transform to adhere to ecological sustainability ( Trivedi et al., 2023). GTI and BDAC have drastically impacted the food business, and further development has been made through artificial intelligence ( AL-Khatib, 2022; Acciarini et al., 2023; Sohu et al., 2024). All of these advancements have the potential to improve sustainable practices through enhanced efficiency across multiple domains, such as energy generation and demand prediction ( Cavazza et al., 2023). In addition, they have a high level of use in reducing waste and increasing effectiveness in matters related to the supply chain ( Frempong et al., 2020). Realizing the growing recognition of sustainability imperatives in food production, it is time to obtain a clear idea of how the manner is to be understood.

The objective of this research is to explore these variables and their relationship with sustainable performance in Pakistan’s food industry. The present study enhances the theoretical concept concerning sustainable practices in the food industry. Identifying the mediated and regulated relationships between these factors supports the improvement of theory-based models in sustainability research. The characteristics of this model make it applicable to many emerging nations and sectors. It also encourages the use of paradigms associated with sustainability and widens the knowledge of sustainable supply chains worldwide. The theoretical developments in this study entail the utilization of the proposed model. This model aims to improve the sustainability of food business in developing countries. These outcomes can be perceived as evidence of the model’s effectiveness. The importance of this study model is that it enables the consideration of other unaddressed sustainability issues in Pakistan’s food production sector. Given the interrelation between the variables, the proposed model provides a more complex view and overview of sustainability measures and their development. To the best of our knowledge, few studies have focused on Pakistan’s global food industry from Pakistani perspectives ( Ali et al., 2020; Ishaq et al., 2023). Thus, there is a need for more quantitative and qualitative studies on interrelated variables and their cumulative impact on Pakistan’s food sector, a developing country marked by challenges and growth opportunities. The significance of the topic of appropriate food business in Pakistan arises because of its relevance to the existing customer needs in planning their diet programs and creating a positive impact towards global sustainability missions on earth ( Oyedijo et al., 2023; Shamsuddoha, 2015).

The following sections of the present research will provide the necessary insights into the global body of scientific literature, methods of carrying out the studies, findings, and their implications for understanding the study frameworks and their outcomes.

## Literature review

The importance of sustainability within Pakistan’s food business has been raised due to rising concerns related to the environment, along with an incline towards fair trade practices from the customer’s point of view. This literature review aims to evaluate the contribution of scholars and knowledge regarding the challenges and opportunities faced by Pakistan’s food industry, considering variables such as GSCM, WM, GTI, BDAC-AI, and SP.

## Theoretical foundations

### Technology acceptance model

The TAM is a theoretical framework that seeks to explain the factors influencing individuals’ adoption and acceptance of new technologies ( Scherer et al., 2019). The TAM mainly considers the individual reasons influenced by peer pressure and the perceived usefulness of the product ( Magsamen-Conrad et al., 2022). Keeping in mind that the core of TAM can be further elaborated to cover organizational fields ( Dhagarra et al., 2019). TAM is a vital model that helps us understand how technological innovations, namely BDAC-AI ( Kraus et al., 2021), combine with the model as a whole. The moderator’s role facing BDAC-AI and SP is placed between managers’ views on how easy AI adoption will be along with the perceived gain of using AI ( Chen, 2023). The link between GSCM-SP and user perspectives in processing food sector of Pakistan embraces moderating role of BDAC-AI, where TAM (Technology Acceptance Model) draws the inner mechanism. Organizations turn to digital methods, exploring the viability and efficiency of doing so in step with GTI and Transformation being considered ( Poláková-Kersten et al., 2023). The topic can be expanded in the sense that the TAM should determine whether businesses see digital technology as a convenient option, which is capable of reducing their expenditures, and whether they are willing to reorganize the structures of their work to meet the required circumstances to be competitive. As a straightforward device, the TAM interface can guide the assessment of whether new technologies can be successfully implemented in any project. TAM has been investigated as part of the potential mechanisms or pathways towards the successful management of the challenges of GTI, BDAC, and the use of AI in organizational contexts ( Wang and Su, 2021).

### Dynamic capabilities theory

In accordance with ( Jiang et al., 2019), the DTC is a process of adjusting, integrating, and rearranging the resources of flexible manufacturing, capability of the organization, and procedures of operation, so the competitive position of the organization will be more prominent and higher productivity will be achieved. Individuals and teams need to be open to learning about organizational change and the use of technology. DTT provides information that is appropriate for organizations planning the growth of technologies that can provide innovation or performance. The model we propose lays great emphasis on the DCT principles that help keep the organizational evolutionary theory running and sustainable, as it encompasses both the continuous adaptation and incorporation of new methods. One of the major points addressed by both GSCM and SP in the field of DCT is the adequate and necessary capabilities of a company to quickly adapt and change its supply chain steps, mostly in the case of external shocks and events ( Ellström et al., 2021). Flexible organizations that inhibit GSCM practices, such as GTI, are fully capable of supporting, controlling, and implementing these practices ( Zhou et al., 2019) and the target of DCT is to develop a wide range of knowledge, and on the basis of that knowledge, how enterprises of the food processing industry of Pakistan can adapt their business ( Kraus et al., 2022). Therefore, these factors are vital for the long-run sustainability of the sector and its competitive edge, in which speed and adaptability are key components ( Ellström et al., 2021).

However, despite the findings of the research stated before, there is a gap when it comes to studying these factors completely within the actual Pakistani food business context among the researchers. The precise functions of trade between the Pakistani food industry were not considered. These involve channels driven by the GTI, WM, and BDAIC AI as mediators and moderators. This gap in recognition still holds even in the presence of literature and information on the subjects of GSCM and SP. The findings of existing studies that are lacking in terms of explaining the underlying mechanisms are insufficient for a comprehensive understanding of how GSCM practices create SP and how this works in the particular context of Pakistan. The wide range of benefits resulting from the development of GSCM has been acknowledged in previous studies ( Debnath et al., 2023; Rupa & Saif, 2022).

In fact, they show up in cost curtailing, efficiency upgrades, and environmental protection measures as long as they are effectively implemented. In the human realm of the food business, the impact and balance of artificial intelligence on the resilience of GSCM, GTI, and WM links is not being communicated properly ( Schintler & McNeely, 2022). It will have worthwhile additions to not just literature but also to the actual applications as an outcome of the analysis of the relationship between GSCM, GTI, WM, BDAC-AI, and SP in the Pakistani food industry. Through this knowledge gap, we aim to understand how sustainable and integral supply chain processes may be implemented in the context of Pakistan. This is set to allow us to provide support to companies or props their initiatives on sustainability improvement, where breakthroughs in technology and innovation can be applied.

The purpose of this study is to add to the constantly developing phenomenon of sustainability practices in the context of a newly industrialized country. The primary goal of the conversation is to provide enterprises, the government, and consumers with useful feedback.

### Sustainable performance

SP is the level of the organization that has been observed to have reached economic, environmental, and social environmental sustainability at the same time as success ( Abu-Rayash & Dincer, 2021). The social responsibility, environmental sustainability, and social effects of modern business are shifting to financial facts ( Kofi Opoku et al., 2023). Such a country would consider this for both ethical and financial reasons ( Ma et al., 2023). One of the goals of the company that made it achieve the SP is to fulfill its financial and social goals, in addition to the prescription of the environment ( Zhang et al., 2023; Kherazi et al., 2024). As a result of this achievement, the organization can be certain that it will remain in business for a long time to come ( Kofi Opoku et al., 2023), and it will stand out in the market ( Peng et al., 2020). SP drops its signature of an unsolicited vocabulary that is relevant to the situations of the poorest nations in the world, as a discovery realized in a research study in this regard ( Castillo-Díaz et al., 2023).

The purpose of this study is to examine the operations of the food processing sector in Pakistan. Furthermore, the study examines whether business operations can make this trade-off effectively between profitability and the pressure that the business pressurizes on the community and the environment. The food-processing industry scores well in contributing to the health and welfare of the nation ( Ashraf et al., 2023b). Thus, business sustainability is required. Several measures, such as eco-friendly practices ( Zhen et al., 2023), minimizing the amount of waste for production ( Hemphill, 2022) and efficiently using the available resources ( Ahmad et al., 2023) must be taken. These initiatives are of even greater relevance to countries such as Pakistan, where this component of development is yet to fully evolve ( Hashmi et al., 2023). Moreover, it is noteworthy that Pakistan personalizes and standardizes its SP food processing industry, which shows that the products being produced are not only risk free but also of premium quality ( Maaz et al., 2021). In such cases, we must remain committed to protecting public and customer health and the innumerable needs of customers.

Several studies have established the presence of SP factors in GSCM strategy planning and operation ( Castillo-Díaz et al., 2023; El Ayoubi & Radmehr, 2023; van der Meulen et al., 2022). It has been documented that SSCM approaches help firms acquire SP for many activities ( Castillo-Díaz et al., 2023; El Ayoubi & Radmehr, 2023). Green supply chain management has received much attention worldwide as a target for sustainable production and consumption planning, especially in Pakistan’s food industry ( Saini et al., 2023b; Das et al., 2023). Sustainable procurement hinges on green supply chain management, which is one of the most crucial components. This bond between the GSCP and SP is very significant for Pakistan’s food industry; it is paramount because of its economic influence and the unique obstacles it overcomes. The links between progress and cleaner environments are critical for attaining sustainable objectives in all sectors. Numerous studies have shown that GSCM practices are financially ( Samad et al., 2021), environmentally, and socially beneficial, as these parameters relate to SP within the food industry ( Yang et al., 2023a). Tostivint et al. (2017) highlight that GSCM can play three main roles: waste lowering, optimal resource use, and system-wide efficiency improvement. For an organization to benefit from cost savings and gain a competitive advantage, the choice to practice GSCM strategies that focus on the procurement of ethically sourced raw materials, waste minimization, and the use of environmentally friendly materials should always be an option ( Li et al., 2022). A Recent study conducted by ( Jalil et al., 2023), cited that implementation of GSCM yields an increase in the social dimension of SP through the creation of favorable working conditions and good treatment of local communities. The provision of GSCM for Pakistan’s food industry has been introduced as the latest solution, which implies achieving a good pace of economic growth while remaining responsible for the environment and society ( Tariq et al., 2023). Companies that work to reduce their carbon footprint are important not only for human society and the environment, but also for increasing their brand equity ( Rehman et al., 2023).

Technologies such as AI and BDAC should be implemented ( Al-Nuaimi et al., 2021), in order to operate SPs in an up-to-date environment ( Belhadi et al., 2020). The management of data using these technologies is a trend. The implementation of technologies, such as AI and bDND, could positively impact the food processing industry in Pakistan ( Khan & Tao, 2022). These tasks represent technologies that have evolved over time and are capable of working with large volumes of data. Thus, they help to identify areas that deserve improvement ( Bresciani et al., 2021). Such technologies are also a blessing for businesses that they should remain in the world according to socially acceptable standards but still earn their income ( Qin et al., 2022), adjusting to the rapidly changing environment of the market ( Morimura & Sakagawa, 2023). The regulation of BDAC-AI provides them with agility and flexibility in restructuring their operations while pursuing the goal of a sustainable future ( Junaid et al., 2022).

### Green supply chain management

GSCM’s main task of GSCM is to integrate the green approach across the entire supply chain ( Chatzoudes & Chatzoglou, 2022). The goal of an energy production scheme is to achieve the best economic performance as well as to reduce waste ( Ali et al., 2019), while also offsetting the adverse environmental effects that arise from supply chain operations such as transportation ( Ghosh et al., 2021a). The field of GSCM comprises fundamental ideas ( Nirmal et al., 2023). The trend of GSCM and other responsible sourcing measures is increasingly being observed in Pakistan’s developing food sector in Pakistan ( Qazi et al., 2022). The application of GSCM strategies might improve supply chain operations, manage both environmental and customer demand factors ( Chatzoudes & Chatzoglou, 2022; Nikseresht et al., 2023), and meet the increasing customer demand for environmentally friendly products ( Ye et al., 2023).

The adaptation of GSCM in the Pakistani food sector has become more widely perceived because of the growing tendency towards environmental protection ( Mubarik et al., 2021) and strictly imposed governmental requirements ( Chatzoudes & Chatzoglou, 2022). Findings from multiple studies show the influence of sourcing from sustainable avenues ( Fallahpour et al., 2021), efficient ( Ghosh et al., 2021b), and inventory management ( Tasnim et al., 2022). Thus, it is imperative that the measures be included when implementing strategies, as they will help in the regulation of the negative environmental impact ( Shi et al., 2022) and efficient utilization of natural resources ( Jha et al., 2021). In Pakistan, the food business is a very important segment of the economy ( Iftikhar et al., 2023); At the same time, it is being challenged by stricter regulations and environmental issues ( Waseem & Rana, 2023). In the context of the logistics industry, organizations are poised to shift to the paradigm of GSCM strategies because of the necessity of maintaining their competitiveness, as outlined by ( Das et al., 2023). Iqbal et al. (2020) introduced some elements of this chain strategy, including but not limited to environmentally sound procurement ( Wang et al., 2020), efficient and lucrative turnaround as far as logistics activities are concerned ( Nikseresht et al., 2023), and the efficient use of resources accompanied by waste minimization ( Yildiz Çankaya & Sezen, 2018; Zuberi & Ali, 2015). These orientations are part of the global aspiration for a cleaner planet and should indeed be a top concern for Pakistan’s food industry in Pakistan ( Ashley, 2016).

The idea of GSCM is an innovative approach that is still in its adolescent stage, which intends to deliberately involve environmental sustainability practices in a variety of aspects of supply chain operations ( Hazen et al., 2020). Recent studies, such as Jabbour et al. (2020), put forward the notion that it is an effective instrument for diminishing the level of carbon emissions, conserving resources ( Ishaq et al., 2023) and amplifying the levels of productivity ( Ali et al., 2020). Within GSCM, components such as responsible inventory management, effective transportation, material reduction in waste ( Rahman et al., 2023), and responsible sourcing ( Trujillo-Gallego & Sarache, 2021) are only a few. Zhu et al. (2019) are convincing that GSCM principles should be added to SC operations. The adoption of these planned measures is fundamental, as it reduces the negative effects that the manufacturing process brings to the environment while increasing the percentage of items that are both sourced and made using ethical means ( Das et al., 2023).

Green procurement methods are considered important in Pakistan’s logistics in Pakistan ( Irfan et al., 2020). Rasheed et al. (2019) stress that orderly procurement requires ethical farming, fair labor, and ecological sourcing. The use of ethical sourcing strategies in the food industry has two significant advantages: improvement in quality and safety ( Asha et al., 2023). Additionally, it increases competition by establishing a higher benchmark for competitors ( DeBoer et al., 2017). The optimization of the transport sector’s operation will be a critical point in the context of GSCM in Pakistan’s food supply chains of Pakistan according to ( Qin et al., 2022). This study, led by ( Ahmed & Sarkar, 2019) revealed that optimizing transportation can play a crucial role in successfully combating carbon emission issues and reducing costs. Hence, this improves the supply chain system and helps meet the targets that state the importance of sustainability factors ( Lopes de Sousa Jabbour et al., 2020).

H1:
*GSCM positively related to SP.*

H2:
*GSCM positively related to GTI.*

H3:
*GSCM positively related to WM.*

## Green technology innovation

Environmental factors are issues that can slowly and surely gain importance in SCM and new product development strategies ( Davidescu et al., 2023). This study investigates the connection between GSCM and GTI, seeking to give them the credit they deserve to promote sustainability ( Sarkis, 2003). GTI actively participates in helping the environment conserve its bits and pieces ( Boye & Arcand, 2013). Implementing and using environment-saving technologies is the most effective way to prevent and mitigate adverse environmental effects. GTI’s success of GTIs depends on the displacement of conventional energy sources with renewable energy sources ( Zuberi & Ali, 2015), the utilization of energy-efficient machinery ( Praveenkumar et al., 2024) and the application of environmentally acceptable building materials ( Hossain & Thomas Ng, 2019). Such technological advancements allow businesses, regardless of size, to benefit, as their environmentally conscious move concurrently enhances their competitive edge ( Rehman et al., 2021).

The sustainability of technology has received particular attention, and many times, sustainable technological advances involving production chains can be observed. A new study by Srivastava and Dey (2018) points to the possibility of implementing technology to foster sustainability of logistics ( Srivastava & Singh, 2020) along the lines of real-time energy consumption monitoring ( Birgonul, 2021) and predictive maintenance of logistical equipment ( Wakiru et al., 2021). The application of GTI stimulates supply chain networks to utilize data-driven policies (( Hassoun et al., 2022) as a result, leading to the reduction of excess means for processes and waste streams ( Hassoun et al., 2022). An important result highlighted in the literature is the existence of an interconnection between GSCM and GTI. For supply chains to be sustainable, GTI offers tools that facilitate this process ( Bukchin & Kerret, 2018), whereas GSCM provides a program that is the basis of this process ( Micheli et al., 2020). When incorporated into organizational operations, these methodologies are heading towards the progress of sustainability ( Saberi et al., 2019; Das et al., 2023). The literature exposes the possibility that GSCM integration with GTI by the organization is a competitive advantage for the business ( Zhang et al., 2022). Söderholm (2020) mentioned that the application of greener supply operations might effectively facilitate company compliance with environmental regulations, decrease running expenses, and attain consumer loyalty that is environmentally ( Hossain & Thomas Ng, 2019). Regarding GSCM and GTI, existing research shows a pronounced correlation between them towards better sustainability in many sectors ( Lerman et al., 2022). The GTI is driven by the use of smart tools and technology in the fields of sustainable procurement ( Rejeb et al., 2023), innovative transport, and waste reduction ( Zuberi & Ali, 2015), focusing on providing a strategic framework for these activities as the next step.

The GSCM strategy for circulating environmental sustainability in supply chain management involves several critical features, including ethical purchasing ( Lim et al., 2023), waste reduction ( Haque et al., 2023), eco-friendly transportation ( Ge et al., 2023), and controlled inventory ( Jauhar et al., 2023). Additionally, the GTI provides technical tools and breakthroughs that allow a cleaner and better way of doing things ( Xie & Teo, 2022). Customers who try to act in accordance with the environmental sustainability value and acknowledge the initiatives that they are by all means proud of. In the event that clients would be willing to undertake more sustainable shopping as well as ensure the ethically correct nature of the products they have bought and the shipping processes they have used ( Ghosh et al., 2021a), the chances of them continuing to do the same is highly probable. The concept of GTI is of significant importance in this context. The implementation of green technical breakthroughs has enabled supply chains to enhance operational efficiency, mitigate emissions, and eliminate waste ( Roy et al., 2023). A recurring outcome of these advancements is the production of ecologically sustainable ( Tasnim et al., 2022). The use of the GTI has the potential to enhance supply chain visibility and traceability as well ( Lai et al., 2023). Therefore, the following hypothesis was developed:

H4:
*GTI is positively related to SP.
*

### Waste management

The long-term viability of Pakistan’s food sector relies on the effective implementation of environmentally friendly WM methods ( Rahman et al., 2023), in accordance with existing worldwide trends, which suggests an increasing focus on waste reduction, recycling, and appropriate waste management practices ( Kayikci et al., 2019). The implementation of effective waste management systems has the potential to yield financial savings and mitigate the environmental impact of organizations ( Hemphill, 2022). As Pakistan, through the process of urbanization ( Ali et al., 2023), experiences population increase and expands its industrial sector ( Ashraf et al., 2023a), the need for viable ways to address the escalating challenges associated with waste buildup becomes more pronounced ( Ashraf et al., 2023b). The importance of effective WM increases as metropolitan areas continue to develop ( Hashmi et al., 2023). WM presents many complex difficulties for a country ( Salam et al., 2023). According to ( Yousafzai et al., 2020), the exacerbation of this problem may be attributed to a lack of public awareness, financial resources, and adequate infrastructure. In densely populated urban regions, the presence of trash streets and public places is visually unappealing and inconvenient for the general population ( Filimonau et al., 2023). Informal waste pickers also provide a significant function in the realm of waste management through their efforts to extract recyclable materials from landfills ( O’Connor, 2021).

In response to these challenges, the government of Pakistan passed a series of legislative measures and undertaken several activities aimed at enhancing the nation’s WM infrastructure ( Yousafzai et al., 2020). One notable government-wide project aimed at promoting cleanliness and educating the public about trash management is the “Clean Green Pakistan” campaign ( Khatibi et al., 2021). Based on the findings of Rasheed et al. (2019) on the 18th Amendment, municipal and state governments are conferred with more powers in waste management, which promotes the use of integrated and economically viable approaches to waste management ( Filimonau et al., 2023) (18th Amendment gives more powers to municipal and state governments; thus, they can implement integrated and economic

Technology is being increasingly applied and innovation is emerging as one of the main tools in Pakistan in battling the ever-growing waste crisis ( Salam et al., 2021). Salam et al. (2023), various smartphone apps that create the best functionalities for the methane management system wipe out the inefficiencies of the entire waste system. Along with the functional consequences of GM on PMM, there are social repercussions. Nowadays, the distribution of natural resources and waste management is a complex issue in which policymakers set up legislative tools to restrict the production process and disposal of harmful materials. Therefore, the application of environmentally sustainable practices has become a prime strategy for businesses ( Mor & Ravindra, 2023). As a result, businesses in the area have moved towards those that incorporate WM in their GSCM plans, according to the study by Kannan et al. (2022). Subsequently, the attainment of standards in sustainable environmental scenarios and world sustainability will be accomplished through the synergistic activity of GSCM and WM practices in developing countries (Sabumon, 2023). The use of WM denotes an inextricable portion of GSCM policy that is geared towards curbing the negative influence of industrialization on this area through this tool ( Haider Naqvi et al., 2023).

A specific growing trend in Pakistan’s waste management sector is evident in the increasing grassroots community involvement and higher social activity levels. Concerns about solid waste collection and disposal as a critical environmental issue in Pakistan have now emerged as a result of many factors ( Ahmad et al., 2023) initially, the increasing levels of economic challenges and decaying infrastructure caused people to face severing opinions on the necessity of taking in innovative strategies as well as passing legislative changes that also include encouraging public participation to effectively address these grave issues ( Yousafzai et al., 2020). Emerging technologies, such as automatic sorting and data analytics systems ( Salam et al., 2023), as well as the process of integrating them, can offer opportunities to enhance waste management in the country ( van der Meulen et al., 2022). In light of the circumstances, it becomes inevitable that the forthcoming studies and policies should have the will to carry on with plan which seeks the contextual as well as sustainable solution. Ultimately, it will prepare the foundation for a sustainable ( Zhen et al., 2023).

H5:
*WM is positively related to SP.*

### Mediation of GTI

In this context, GTI is a general strategy model that promotes new clean technologies and hitches them with appropriate attitudes in different sectors ( Punj et al., 2023). It focuses on the food and beverage industry ( O’Connor, 2021). The generation and development of the GTI can be due to an appreciation of ecological and environmental sustainability, together with the fact that climate challenges need immediate intervention ( Hassoun et al., 2022). Innovations in these fields cover an extensive range of topics, such as energy-saving machines ( Xie & Teo, 2022), green production methods ( Lai et al., 2023), environmentally friendly packaging ( Hong et al., 2018), and product design, which has no influence on the environment ( Peng et al., 2020). Nevertheless, the GTI is not just a technological development ( Lepore et al., 2023). It incorporates new business strategies that focus on environmental gain, optimization of resource efficiency, and ensuring the long-term commercial sustainability of organizations ( Alyahya et al., 2023; Yousafzai et al., 2020).

The accomplishment of the food processing industry against SP without aid from the GTI may be extensive ( Alyahya et al., 2023). Energy conservation using sustainable technologies; green production and packaging; all of these developments hold very bright prospects for food processing industries as they are concerned with the carbon footprint and resource efficiency of the food processing firms ( Yang et al., 2023b). The employment of GTI is a way to meet the growing needs of conscientious customers who are environmentally friendly are met ( Xie & Teo, 2022). As a result, it not only increases market share but also investor value, which is measured by return on equity, return on invested capital, and cash flow ( de Burgos-Jiménez et al., 2013). Moreover, the highly inclusive green practice promotes a company that has been able to sustain itself because it is able to fit various market conditions and sustainability issues ( Haider Naqvi et al., 2023). The successful implementation of GTI into operational processes indeed facilitates the promotion of sustainability ( Galanakis et al., 2021), reduction of ecological impact ( Filimonau et al., 2023), and Sustainable Performance ( Ma et al., 2023). Overall, such results are key to the competitiveness of the industrial sector and to achieving environmental targets that are valid around the world ( Ren et al., 2023).

In view of their efforts to create sustainability, enterprises that operate in these industries are more likely to implement GTI ( Ma et al., 2023). The exploitation of the GTI could be the most potential feature that plays a role in the evaluation of operations management ( Zhang et al., 2023). A previous study by Maaz et al. (2021) showed us the same aim. These technologies are particularly suitable for managing this area because they are designed to deal with many nuances ( Ashaari et al., 2021) . BDAC-AI provides solutions that help to concentrate on control and supervision ( Alyahya et al., 2023), of the information on how well environmentally friendly measures are being used or implemented by organizations. The benefit of decision-making on a data basis is important for the realization of GTI goals because it helps align tech investments with sustainability purposes ( Hassoun et al., 2022). The joint venture between the GTI and the food processing sectors of low-income countries is the BDAC-AI initiative targeting the gulf ( Al-Nuaimi et al., 2021). It holds a hegemonic position in the global innovation market, so its prime task is to ensure that modified solutions provided by the GTI are targeted to the peculiarities of these countries, such as opportunities and difficulties ( Cheng et al., 2023).

H7a
*GTI significantly mediates the relations between GSCM and SP.*

H7b
*WM significantly mediates the relations between GSCM and SP.*

### Big Data Analytics capabilities-Artificial intelligence (BDAC-AI)

In addition, BDAC-AI has the ability to carry out instant adjustments to any change in the market and to any customer preference changes ( Bag et al., 2023; Hongyun et al., 2023; Sohu et al., 2023). The integration of BDAC-AI technology is the only method through which food processing companies can properly deal with environmental sustainability of the environment ( Zhen et al., 2023) and health concerns ( Ashraf et al., 2023b). This is achieved through the development of RT logistics and product propositions, along with the establishment of supply chain activities ( Bag et al., 2023). The fact that the capacity for change and adaptability among its members needs to be employed as a crucial determinant of the long-term success of the firm ( Agyabeng-Mensah & Tang, 2021) is a definitive factor instrumental in the long-term success of the firm. The arrival of green technology has played a part in initiatives aimed at keeping the planet environmentally friendly, in this way promoting the conservation and careful use of limited resources such as water, energy, land, and wildlife ( Calza et al., 2020), Ren et al., 2023). The BDAC-AI system plays the role of this correlation, which is known as data-driven decision-making and enables market players to adapt to dynamic market environments ( Ashaari et al., 2021).

Sustainability of agriculture in Pakistan has become a vital area of concern for the agricultural sector, which is actively interested in recent ideas of sustainability ( Gupta et al., 2020). The presence of the private sector is essential to achieving the fundamental objectives of the SDP in Pakistan’s food ( Ashraf et al., 2023b; Yousafzai et al., 2020). GTI aims to be a pathfinder organization that doctrines eco-friendly technologies and methods to help target Pakistani businesses achieve their sustainability objectives ( Raeesi et al., 2023). As Pakistan is experiencing prevailing circumstances, we need to ensure that BDAC-AI will be essential Ashaari et al. (2021). Bag et al. (2020) wrote on the importance of BDAC-AI in that business companies are supplied with the necessary anchors for monitoring and evaluating the use and effectiveness of environmentally sustainable technology equipment. Based on the results of ( Belhadi et al., 2020; Dakhan et al., 2020), many places have invested in technology while achieving sustainability targets efficiently and effectively.

H6:
*BDAC-AI is positively related to SP.*

H8:
*BDAC-AI significantly moderates the relations between GTI and SP*

## Method

### Research design

**Figure 1.**
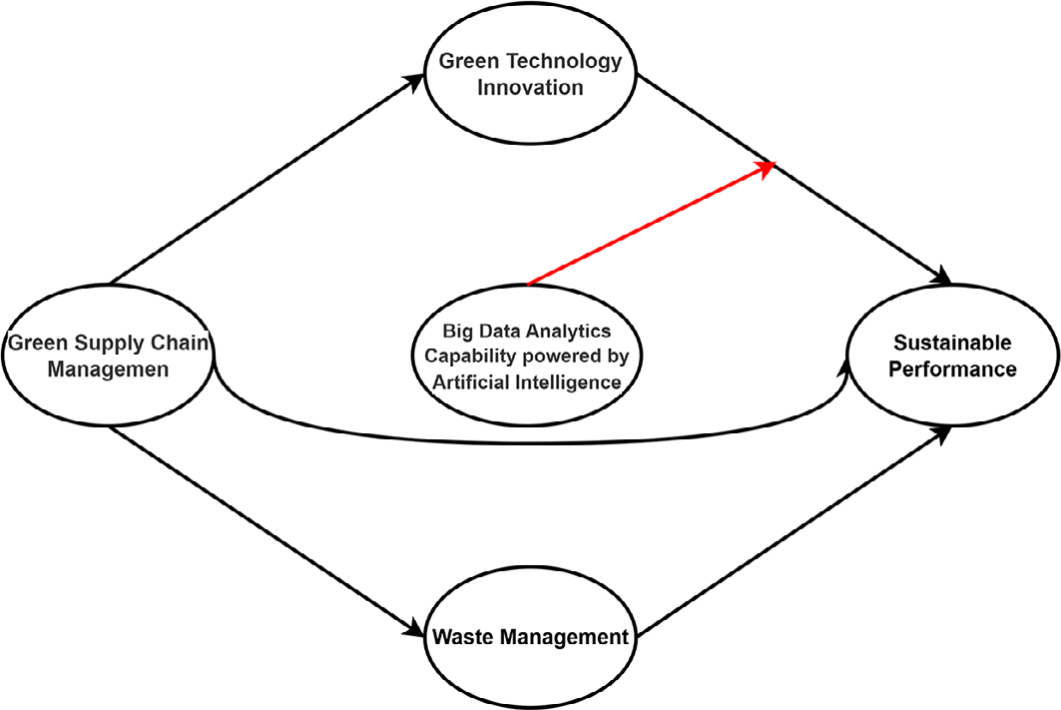
Theoretical framework. Source: Authors’ calculations.

In this study, a quantitative approach was adopted because it suggests that this approach is suitable for understanding the relationships between variables through complex models. The independent variable, green supply chain management; two mediating variables, including waste management and green technology innovation; and one moderating variable, big data analytics capacity-artificial intelligence, were tested in this study. The conceptual model illustrating the intricate relationships among the variables is presented in Figure 1. The conceptual model places BDAC-AI as a moderator between GTI and SP due to its strategic role in enhancing the efficiency and implementation of green technologies. GSCM influences both GTI and WM, which subsequently affect SP. This structure reflects the principles of the Technology Acceptance Model (TAM) and Dynamic Capability Theory (DCT), offering a robust explanation for the interconnected pathways toward sustainability.

### Ethics and consent

This study “Impact of Green Supply Chain Management on Sustainable performance: A dual mediated-moderated analysis of Green Technology Innovation and Big Data Analytics Capability powered by Artificial Intelligence” was approved by the Ethics Committee of The University of Okara, which constitutes the departmental Ethics Approval Committee (REBSSH/2023/2-7) on July 04, 2023. All informants provided written and oral informed consent to participate in this study.

### Procedure of data collection

In this study, food manufacturing firms and those with managerial positions in Pakistan were targeted. Four hundred ninety-five cases were used for data analysis, and the sampling strategy was a random sampling strategy to give all employees of the stated firms a chance. The individuals’ personal information was kept confidential, and all individuals were provided with a consent form before data collection. Furthermore, in this study, a structured, closed-ended questionnaire was distributed among manufacturing SMEs in food processing in Pakistan. They were requested to fill the form through Google Forms, which took one and two weeks.

Additional details about the participating firms are as follows: The firms involved in this study qualified as SMEs according to the definition by the State Bank of Pakistan, which defines SMEs as enterprises with fewer than 250 employees and an annual turnover not exceeding PKR 800 million. The sampled companies varied in age, ranging from 5 to 35 years of operation, and included both privately owned and family-owned firms. Most firms had 2–5 regional branches or subsidiaries within Pakistan. The average number of employees per firm was approximately 110. The participating firms reported annual revenues between PKR 50 million and PKR 700 million.

Clarification: Although the questionnaire was distributed broadly using a random sampling strategy within the selected firms, only managerial-level employees were asked to complete the responses. Thus, all data collected originated from managers with strategic or operational responsibilities.

### Data analysis

The quantitative approach helped analyze the data gathered through various statistical tools. The present research employed structural equation Modelling (SEM) because it supports the study of the hypotheses regarding the relationship among the variables ( Hair et al., 2021). SEM facilitated the simultaneous analysis of the complicated relationships within the study. However, at this start, SPSS was used to clean the data and deal with missing values. Subsequently, a study using SEM was performed, followed by applying Smart PLS (Partial Least Squares) to determine the correlations among the variables. Specifically, any response missing more than 10% of items was excluded, while minimal missing data was handled via mean substitution.

The methodology chosen aligns with the primary aim of this study. The use of a carefully constructed questionnaire facilitated the acquisition of comprehensive and detailed responses ( George, 2019) from the executives of the respective companies. The hypothesized associations were thoroughly investigated using quantitative methodology and SEM. Nevertheless, the quantitative approaches and other procedures used may have been insufficient in terms of statistical seriousness, limiting the ability to establish reliable correlations and to present a more comprehensive analysis. Owing to the inherent quantitative character of the research issue, statistical analysis was used to quantify the connections, whereas a survey-based technique was considered the most suitable approach.

### Scale development

To measure the above-stated variables, instruments were adopted from past studies published in reputable journals, ensuring reliability and validity. The six research items of green supply chain management were adopted from study of ( Le, 2023), who developed these items based on a comprehensive review of the literature and validated them through expert feedback and pilot testing. The second variable, green technological innovation, consists of five research items taken from ( Sahoo et al., 2023). The third variable, waste management, was obtained from the study ( Obuobi et al., 2022). Fourth variable big data analytics capacity-artificial intelligence four items are adopted from study ( Benzidia et al., 2021). Finally, sustainable development was taken from the research of Le et al. (2022) and Tjahjadi et al. (2022) with six items. These items were formulated based on an extensive literature review and validated through confirmatory factor analysis (CFA) to ensure their accuracy and relevance. Most studies used rigorous methodologies, including pilot studies and validation through structural equation modeling, to establish the scales’ validity and reliability.

## Results and discussions

### Testing for convergent and discriminant validity

Factor loadings indicate the strength of the relationship between each question and its respective constructs (such as BDAC-AI, GSCM, GTI, SP, and WM). Higher item loadings signified stronger relationships. Cronbach’s alpha (α) is a measure of internal consistency. Higher values (α > 0.7) indicated good reliability, suggesting that the items within each construct reliably measured the same underlying concept. Similar to α, Composite Reliability (CR) measures the internal consistency reliability, and a CR value > 0.7 Shmueli et al. (2019) is considered acceptable, indicating that the items reliably measure their respective constructs ( Hair et al., 2021). An AVE above 0.5 suggests that more than 50% of the variance is captured by the construct, indicating good convergent validity. The outer variance inflation factor (VIF) checks for multicollinearity among the items within a construct, and the inner VIF represents multicollinearity between variables. VIF Values of < 5 are generally acceptable, indicating that multicollinearity is not problematic.

Table 1 highlights factor loadings, α, and CR values > 0.7, indicating robust relationships between the items and their respective constructs. The items reliably measure their constructs, and indicate consistency in measuring the constructs ( Hair et al., 2019). AVE values >0.5 show that a significant amount of variance is explained by the variables, indicating good convergent validity. VIF values <5 indicated no significant multicollinearity issues among the items within each construct.

**Table 1.** Reliability and validity.

Items	loadings	Variables	Alpha	CR	AVE	Out VIF
BDAC-AI1	0.818	BDAC-AI	0.833	0.888	0.666	1.780
BDAC-AI2	0.812					1.760
BDAC-AI3	0.815					1.817
BDAC-AI4	0.819					1.903
GSCM1	0.781	GSCM	0.897	0.921	0.661	2.221
GSCM2	0.821					2.500
GSCM3	0.867					3.303
GSCM4	0.790					2.407
GSCM5	0.779					2.031
GSCM6	0.836					2.784
GTI1	0.844	GTI	0.886	0.916	0.687	2.663
GTI2	0.834					2.595
GTI3	0.857					2.899
GTI4	0.766					1.790
GTI5	0.841					2.640
SP1	0.819	SP	0.894	0.919	0.654	2.559
SP2	0.800					2.466
SP3	0.798					2.777
SP4	0.814					2.623
SP5	0.824					2.682
SP6	0.796					2.806
WM1	0.816	WM	0.872	0.907	0.661	2.071
WM2	0.824					2.249
WM3	0.806					2.023
WM4	0.806					1.956
WM5	0.813					2.295

Source: Authors’ calculations.

### Discriminant validity

The Heterotrait-Monotrait Ratio of Correlations (HTMT) and the Fornell and Larcker (FnL) criteria were used to assess discriminant validity in this study. In the present study, two critical values, the HTMT and the Fornell and Larcker criteria, helped determine discriminant validity ( Henseler et al., 2015). The values are shown in Table 2 below; all values are less than the suggested 0.90 ( Hair et al., 2019). The HTMT values ranged from 0.617 to 0.897, and the FnL diagonal values are the square root of AVE, indicating no issue in discriminant validity.

**Table 2.** Discriminant validity and correlations.

	BDAC-AI	GSCM	GTI	SP	WM
**HTMT Ratio of Correlations **					
BDAC-AI					
GSCM	0.705				
GTI	0.828	0.668			
SP	0.897	0.778	0.896		
WM	0.749	0.617	0.684	0.808	
**Fornell and Larcker Criterion **					
BDAC-AI	0.816				
GSCM	0.615	0.813			
GTI	0.717	0.603	0.829		
SP	0.776	0.703	0.799	0.809	
WM	0.640	0.551	0.606	0.717	0.813

Source: Authors’ calculations.

### VIF and F Square

**Figure 2.**
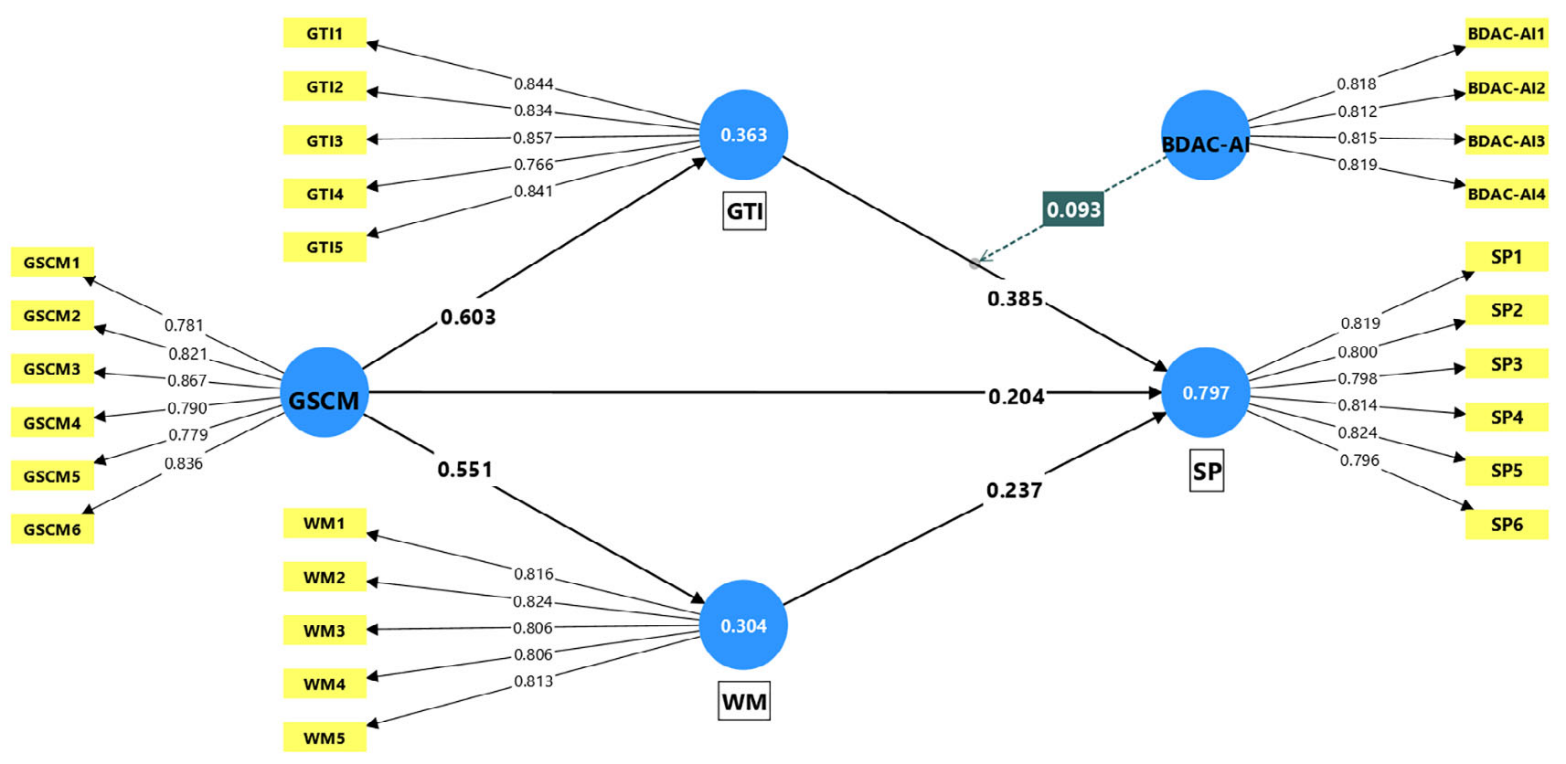
Measurement model. Source: Authors’ calculations.

**Figure 3.**
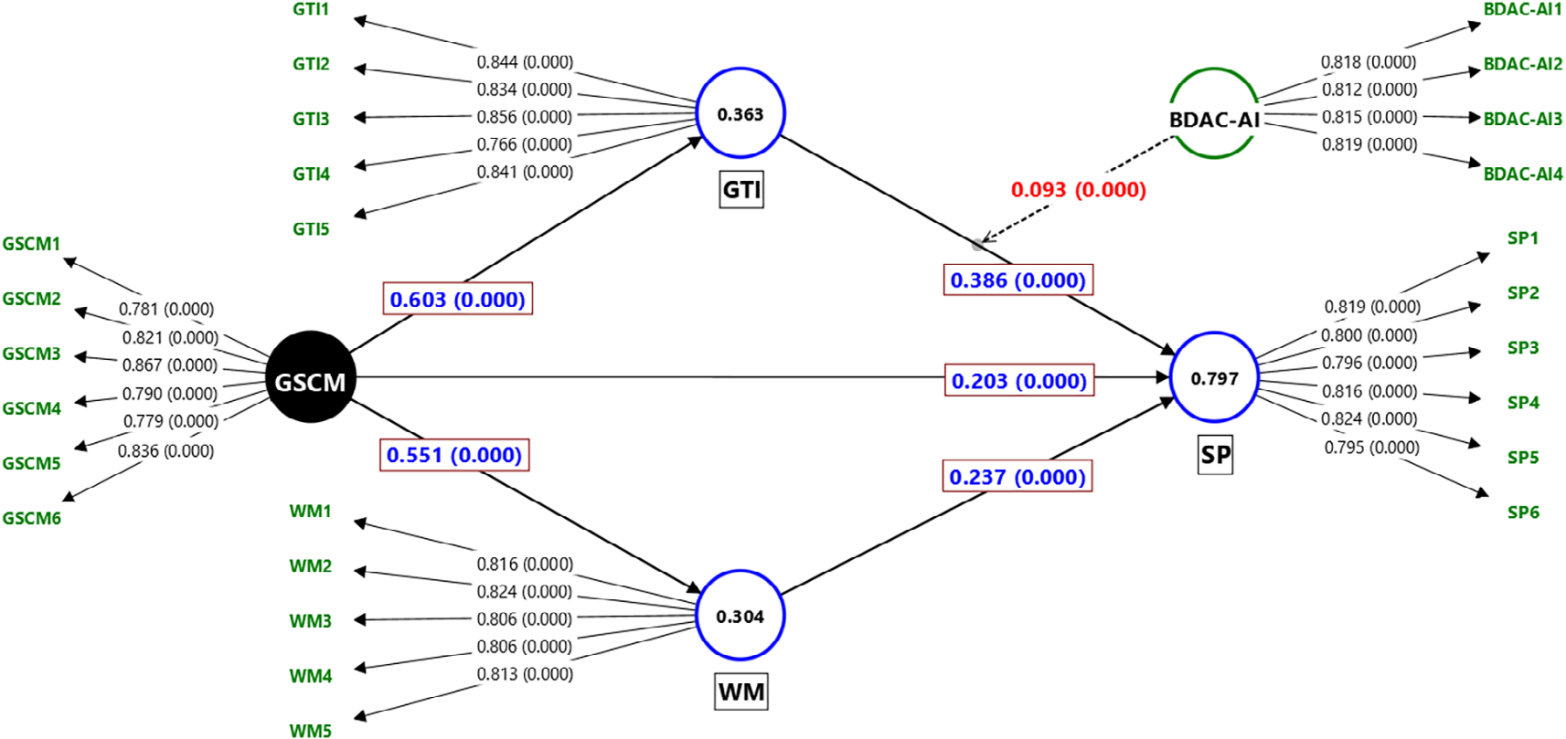
Structural Model (At 10,000 Bootstrapping). Source: Authors’ calculations.

F-Square is used to measure the proportion of variance explained in the dependent variable or effect size by adding a specific independent variable to the model. This helps to understand how much of the variance in the endogenous variable is accounted for by the inclusion of the exogenous variable. Higher F-Square values indicate that a larger proportion of the variance in the higher-order construct can be explained by its constituent variables. Table 3 shows that the F Square values for GTI and GSCM are 0.570. The remaining f-squared values are listed in Table 3. The inner VIF and F Square values suggest that multicollinearity is not an issue in the model, and all constructs are well explained by their constituent variables, which adds to the credibility of this research model. The measurement model is depicted in Figure 2. The structural model is illustrated in Figure 3.

**Table 3.** VIF and F Square.

	GTI	SP	WM
**Variance inflation factor **			
BDAC-AI		2.560	
GSCM	1.000	1.837	1.000
GTI		2.421	
WM		1.916	
**F Square**			
BDAC-AI		0.100	
GSCM	0.570	0.112	0.437
GTI		0.300	
WM		0.144	

Source: Authors’ calculations.

### R and adjusted R square

The model’s goodness of fit was evaluated by the values of R-squared (R ^2^) and Adjusted R-Squared (R ^2^ adjusted). They provide information on how well the independent variables explain the variance in the dependent variable. Along with R ^2^, Q ^2^, SRMR, and NFI values were used to predict the goodness of fit of the model. Table 4 shows that the SRMR and NFI values are 0.061 and 0.802, respectively, which suggests that the model offers a good fit for the data analysis. The explanatory power of the model was quantified by calculating its R ^2^ value. For the GTI, R ^2^ is 0.363, which suggests that approximately 36.3% of the variance in DP is explained by the GTI in the model. All other values are listed in Table 4.

**Table 4.** R-square and Q-square.

	R-square	R-square adjusted
GTI	0.363	0.362
SP	0.797	0.795
WM	0.304	0.302

	Q ^2^predict	RMSE
GTI	0.360	0.803
SP	0.607	0.629
WM	0.301	0.840
SRMR	0.061	
NFI	0.802	

Source: Authors’ calculations.

In summary, both R ^2^ and R ^2^ adjusted values for all constructs are relatively high, suggesting that a considerably large amount of variance in the endogenous constructs (SP, WM, and GTI) is explained by the exogenous variables in the current model, which indicates that the model provides a reasonably good fit to the data and supports the relationships between the variables. To evaluate the predictive power of the model, researchers used the Q ^2^ value technique ( Shmueli et al., 2019). Q ^2^ determines the extent to which an independent variable influences the dependent variable in the model. For the current study, the Q ^2^ values of GTI and WM are 0.360 and 0.301, respectively, which implies a substantial predictive importance of the depedent6 variables.

### Direct path analysis

A direct path analysis examines the direct relationships between exogenous and endogenous constructs. All hypotheses (H1–H6) were supported, showing significant relationships. In Table 5, H1 suggests that GSCM has a positive effect on the GTI, and the T-value (22.781) and P-value (significant at 0.000) confirm that this relationship is significant. Similarly, H5 shows that WM has a positive effect on SP, supported by a T-value of 7.831 and a significant p-value (0.000).

**Table 5.** Direct path analysis.

	*Hypotheses*	Path	Mean	SD	T Value	P value	*Decision*
**Direct path analysis **							
*H1*	*GSCM -> GTI*	0.603	0.603	0.026	22.781	0.000	*Acc*
*H2*	*GSCM -> WM*	0.551	0.553	0.029	19.010	0.000	*Acc*
*H3*	*GSCM -> SP*	0.203	0.203	0.027	7.445	0.000	*Acc*
*H4*	*GTI -> SP*	0.386	0.385	0.039	9.825	0.000	*Acc*
*H5*	*WM -> SP*	0.237	0.237	0.030	7.831	0.000	*Acc*
*H6*	*BDAC-AI -> SP*	0.228	0.229	0.033	6.917	0.000	*Acc*
**Specific indirect path analysis/mediation **							
*H7a*	*GSCM -> GTI -> SP*	0.232	0.232	0.026	9.019	0.000	*Acc*
*H7b*	*GSCM -> WM -> SP*	0.131	0.131	0.019	6.977	0.000	*Acc*
**Total indirect path analysis **							
	*GSCM -> SP*	0.363	0.363	0.027	13.374	0.000	*Acc*
**Moderation **							
*H8*	*BDAC-AI x GTI -> SP*	0.093	0.094	0.021	4.454	0.000	*Acc*

Source: Authors’ calculations.

### Specific indirect path analysis/mediation

Specific indirect path analysis examines the mediating effect of constructs on the relationships between exogenous and endogenous constructs. The GTI and WM play mediating roles in the relationship between GSCM and SP. All the mediating paths (H7a–H7b) were positive and significant. H7a and H7b show that the mediating effect of GSCM on SP through GTI and WM is positive and significant (T-value: 9.019, p-value: 0.000, T-value: 6.977, P-value: 0.000). Here, the total indirect path analysis suggests partial and full mediation. All indirect paths are significant, so all mediating relations are partially mediated.

## Moderation

Finally, the researchers examined the moderating role of environmental dynamism. The moderating effect of H8 is significant and positive. Table 5 shows that the interaction between BDAC-AI and GTI significantly affected ECP (T-value: 4.454, P-value = 0.000). These findings support Hypothesis H8. Therefore, environmental dynamism enhances the positive impact of BDAC-AI and GTI on ECP, highlighting its crucial role in achieving sustainable performance.

## Discussion and concluding remarks

The findings from the empirical analysis provide important insights into the relationships among GSCM, GTI, WM, BDAC-AI, and SP within the food processing multinational organizational context within Pakistan. There was a positive and significant relationship between GSCM and GTI, WM, and SP. This implies that an emphasis on GSCM practices is positively associated with fostering GTI, Waste Management strategies, and overall environmental performance within organizations. The mediated paths indicate partial mediation of GTI and WM in the relationship between GSCM and SP. These findings underscore the importance of intermediary processes in translating GSCM practices into enhanced sustainability outcomes. Additionally, the moderation effect of BDAC-AI on the relationship between GTI and SP demonstrates the influence of advanced analytics capabilities augmented by Artificial Intelligence in moderating the impact of GTI on SP.

The present study’s findings confirm the direct effects of GSCM, GTI, and WM on the sustainable performance of SME manufacturing in the food processing sector in Pakistan. Furthermore, TAM and DCT theories suggested these variables, and their relationship was confirmed in the present study. However, the unique finding of the present study is that green technological innovation was found to have a more significant and positive impact on sustainable performance due to higher beta values than other variables in this study. Furthermore, the indirect effect of GTI between GSCM and SP was found to have a more significant impact on SP compared to the indirect effect of WM, due to higher path coefficients. Finally, the moderating role of BDAC-AI must be addressed in the present study. This moderates the relationship between GTI and SP.

### Theoretical and practical implications

In the present study, two TAM and DCT supported the variables of the study, including GSCM as an independent variable, two mediating variables (GTI and WM), and one moderating variable (BDAC-AI) on SP in SMEs manufacturing food supply chain processing firms in Pakistan, a developing country. TAM supports technology acceptance in today’s business environment, where environmental protection is a key concern. Similarly, the DCT confirmed responsiveness towards environmental protection and adaptability within the organization. Policymakers and top management of SME manufacturing in the context of the food processing sector in Pakistan can adopt these variables in their future strategies. They can benefit from using scarce resources and better productivity within firms.

While the study confirms the effectiveness of GSCM, GTI, and BDAC-AI in enhancing sustainable performance, firms often face practical barriers in implementing such strategies. Financial constraints, limited access to advanced technology, lack of trained personnel, and resistance to organizational change are commonly reported challenges, particularly among SMEs. Addressing these barriers may require internal capacity-building and external support through public-private partnerships or sector-specific training initiatives.

### Policy implications

To accelerate the adoption of GSCM practices and digital innovations like BDAC-AI, supportive government policies are essential. These may include tax incentives for green investments, subsidies for waste management infrastructure, skill development programs in green technologies, and national guidelines for AI-driven sustainability practices. Such initiatives can empower SMEs in the food sector to transition more effectively toward holistic sustainability performance.

## Limitations and future research directions

Along with a few contributions, the present study has certain limitations. Data limited to a cross-sectional approach in future longitudinal data types can be collected to validate the existing findings. This study was limited to two theories: TAM and DCT. However, other theories that support the current conceptual model can be tested in the future. This is limited to food-processing manufacturing SMEs in Pakistan. Other sectors, such as textiles and pharmaceuticals, will be considered in the future.

The present study is context-specific, focusing solely on the food processing sector in Pakistan. While this provides depth and relevance to sector-specific sustainability challenges, it limits the generalizability of findings to other industries. Future studies are encouraged to replicate this model in sectors such as textiles, manufacturing, or pharmaceuticals. Additionally, cross-country comparisons in similar developing economies may offer richer insights into regional differences in GSCM practices and sustainability outcomes.

As with most survey-based research, this study relies on self-reported responses from managerial personnel. Although measures were taken to ensure confidentiality and encourage honest answers, there remains a possibility of social desirability bias. Future research should consider incorporating objective performance metrics, third-party sustainability audits, or cross-verification with company records to strengthen data validity and reduce reliance on subjective assessments.

This study utilized a cross-sectional design, which captures relationships at a single point in time. As such, it may not fully reflect the long-term impact of GSCM practices, GTI adoption, or the moderating role of BDAC-AI on sustainability performance. Future longitudinal studies could provide more nuanced insights into how these relationships evolve and the presence of lagged effects.

### Ethics and consent

This study “Impact of Green Supply Chain Management on Sustainable performance: A dual mediated-moderated analysis of Green Technology Innovation and Big Data Analytics Capability powered by Artificial Intelligence **”** was approved by the Ethics Committee of The University of Okara constitutes the departmental Ethics Approval Committee (REBSSH/2023/2-7) on July 04, 2023. All informants provided written and oral informed consent to participate in this study.

## Data Availability

Figshare: Dataset & Questionnaire: DOI: https://doi.org/10.6084/m9.figshare.26247548 ( Junejo, 2024) This project contains the following underlying data:
• Dataset. csv. Dataset • Final Questionnaire.docx Data are available under the terms of the Creative Commons Attribution 4.0 International license (CC-BY 4.0).

## Software availability

The primary software used for data analysis in this study includes SmartPLS and SPSS. SmartPLS offers a 30-day free trial and can be accessed at http://www.smartpls.com. SPSS is a proprietary software available from IBM at https://www.ibm.com/products/spss-statistics .

For researchers seeking free and open-source alternatives, the following software can be used:

• WarpPLS for PLS-SEM, available at http://www.warppls.com.
• JASP for general statistical analysis, available at https://jasp-stats.org.
• R for comprehensive statistical analysis, available at https://www.r-project.org.
