# Supplementary material for: Impact of Green Supply Chain Management on Sustainable Performance: A Dual Mediated-moderated Analysis of Green Technology Innovation And Big Data Analytics Capability Powered by Artificial Intelligence
Source: F1000Res. 2025 Sep 8;13:1140. Originally published 2024 Oct 7. [Version 2] doi: 10.12688/f1000research.154615.2 (PMC12504947; doi:10.12688/f1000research.154615.2)
Supplement: Peer review history

# Reviewer report: Reviewer response for version 2

**DOI:** 10.5256/f1000research.186427.r412364

**Published:** 2025-10-07

**Authors:** Samir Zic

**Affiliations:** 1 University of Rijeka, Rijeka, Croatia

No comments.

Is the work clearly and accurately presented and does it cite the current literature?

Yes

If applicable, is the statistical analysis and its interpretation appropriate?

I cannot comment. A qualified statistician is required.

Are all the source data underlying the results available to ensure full reproducibility?

Partly

Is the study design appropriate and is the work technically sound?

Partly

Are the conclusions drawn adequately supported by the results?

Partly

Are sufficient details of methods and analysis provided to allow replication by others?

Partly

Reviewer Expertise:

Industrial management, Inventory optimization, Supply chain optimization, Operational research

I confirm that I have read this submission and believe that I have an appropriate level of expertise to confirm that it is of an acceptable scientific standard.

# Reviewer report: Reviewer response for version 2

**DOI:** 10.5256/f1000research.186427.r412894

**Published:** 2025-09-30

**Authors:** Samera Nazir

**Affiliations:** 1 Xi'an Fanyi University, Xi'an, Shaanxi, China

**Improve the figures quality**

- Review your graphs, charts, and diagrams to ensure they are **clear, high resolution, and professional**.
- Use consistent **fonts, colors, and labels**.
- Replace blurry images with vector-based charts (made in Excel, SPSS, R, or similar tools).
- Ensure axes, legends, and captions are **readable** and follow journal guidelines
- Ensure you **cite recent studies (2021–2025)**, not just older ones.
- Integrate recent **journal articles, systematic reviews, or industry reports** to show that your work is built on the latest developments.
- Highlight gaps identified in **current literature**, and show how your study addresses them.

Is the work clearly and accurately presented and does it cite the current literature?

Yes

If applicable, is the statistical analysis and its interpretation appropriate?

Yes

Are all the source data underlying the results available to ensure full reproducibility?

Yes

Is the study design appropriate and is the work technically sound?

Yes

Are the conclusions drawn adequately supported by the results?

Yes

Are sufficient details of methods and analysis provided to allow replication by others?

Yes

Reviewer Expertise:

Its good

I confirm that I have read this submission and believe that I have an appropriate level of expertise to confirm that it is of an acceptable scientific standard, however I have significant reservations, as outlined above.

# Reviewer report: Reviewer response for version 1

**DOI:** 10.5256/f1000research.169663.r342802

**Published:** 2025-01-03

**Authors:** Samir Zic

**Affiliations:** 1 University of Rijeka, Rijeka, Croatia

Study design follows positive practice and established routines in scientific literature. The authors have provided enough recent studies as references.

 The authors mentioned that “food manufacturing firms and those with managerial positions in Pakistan were targeted.” An explanation of important aspects of participating companies, such as size (number of employees), maturity, years in business, financial results, number of subsidiaries, etc., is not provided and is an important aspect of the research. By reading this paper in its current form, readers can't find more details about companies that participated in this research (except they are classified as SMEs, but that classification changes from country to country. The authors didn’t provide information about Pakistan’s definition of SME). This is an important aspect and needs to be addressed appropriately. With a lack of information about participating companies, research can’t be repeated outside of Pakistan or even within Pakistan but in different regions.

 Variables have different names in Excel file and text. It should be uniform.

 The authors state, “SPSS was used to clean the data and deal with missing values”. This process affects the results and outcomes of the study, but information on the number of missing values and the cleaning process is not provided.

 We don’t know how many questionnaires were sent and how many were received.

 In the text, the authors mention 495 responses from managers in the food processing industry; however, only 492 are listed in Excel.

 “In this study, food manufacturing firms and those with managerial positions in Pakistan were targeted. Four hundred ninety-five cases were used for data analysis, and the sampling strategy was a random sampling strategy to give all employees of the stated firms a chance.” In the end, who did fulfill the questionnaire – managers or “all employees of the stated firms”?

 The questionnaire has 5 variables with a total of 26 questions. Each question can be answered with values of 1 to 5. This is not clearly defined within the paper. Results from 492 (based on Excel) or 495 (based on paper) questionnaires can have a cumulative value from 26 up to 130. Having identical cumulative values for different answers is not uncommon. In this research, based on 492 results, there are 26 groups with a cumulative value of 71, 18 groups with a cumulative value of 76 etc.).

 However, out of 492 questionnaires, 65 (13%) have identical answers on all 26 questions, which is challenging to explain statistically. Furthermore, some of these 65 duplicate questionnaires occur in larger groups. This is noticeable for questionnaires with a cumulative value of 65, where the same questionnaire repeats 8 times. Statistically, there is a very small chance of something like that. Authors are invited to explain or correct this. This is a crucial question since further analyses and conclusions arise from this data.

 An explanation of the conceptual model, as presented in Figure 1, is not provided. The authors didn’t provide info on why they are connected to the framework as they are. Positioning Big Data Analytics Capability – Artificial Intelligence on different positions seems reasonable but would completely change the outcomes of this research. More explanations are required.

Is the work clearly and accurately presented and does it cite the current literature?

Yes

If applicable, is the statistical analysis and its interpretation appropriate?

I cannot comment. A qualified statistician is required.

Are all the source data underlying the results available to ensure full reproducibility?

Partly

Is the study design appropriate and is the work technically sound?

Partly

Are the conclusions drawn adequately supported by the results?

Partly

Are sufficient details of methods and analysis provided to allow replication by others?

Partly

Reviewer Expertise:

Industrial management, Inventory optimization, Supply chain optimization, Operational research

I confirm that I have read this submission and believe that I have an appropriate level of expertise to confirm that it is of an acceptable scientific standard, however I have significant reservations, as outlined above.

# Response

**Published:** 2025-08-20

**Authors:** Faisal Ejaz

**Affiliations:** Internaitonal Relations, Minhaj University Lahore, Lahore, Punjab, Pakistan

**Reviewer 1 comments**

**Comment 1a:** **The authors mentioned that “food manufacturing firms and those with managerial positions in Pakistan were targeted.” An explanation of important aspects of participating companies, such as size (number of employees), maturity, years in business, financial results, number of subsidiaries, etc., is not provided and is an important aspect of the research. By reading this paper in its current form, readers can't find more details about companies that participated in this research (except they are classified as SMEs, but that classification changes from country to country. The authors didn’t provide information about Pakistan’s definition of SME). This is an important aspect and needs to be addressed appropriately. With a lack of information about participating companies, research can’t be repeated outside of Pakistan or even within Pakistan but in different regions.**

**Response:** **Additional details about the participating firms are as follows: The firms involved in this study qualified as SMEs according to the definition by the State Bank of Pakistan, which defines SMEs as enterprises with fewer than 250 employees and an annual turnover not exceeding PKR 800 million. The sampled companies varied in age, ranging from 5 to 35 years of operation, and included both privately owned and family-owned firms. Most firms had 2–5 regional branches or subsidiaries within Pakistan. The average number of employees per firm was approximately 110. The participating firms reported annual revenues between PKR 50 million and PKR 700 million.**

**Comment 1b:** **Variables have different names in Excel file and text. It should be uniform.**

**Response:**

**Comment 1c:** **The authors state, “SPSS was used to clean the data and deal with missing values”. This process affects the results and outcomes of the study, but information on the number of missing values and the cleaning process is not provided.**

**Response:** **Specifically, any response missing more than 10% of items was excluded, while minimal missing data was handled via mean substitution.**

**Comment 1d:** **We don’t know how many questionnaires were sent and how many were received. In the text, the authors mention 495 responses from managers in the food processing industry; however, only 492 are listed in Excel.**

**Response:**

**Comment 1e:** **“In this study, food manufacturing firms and those with managerial positions in Pakistan were targeted. Four hundred ninety-five cases were used for data analysis, and the sampling strategy was a random sampling strategy to give all employees of the stated firms a chance.” In the end, who did fulfill the questionnaire – managers or “all employees of the stated firms”?**

**Response:** **Clarification: Although the questionnaire was distributed broadly using a random sampling strategy within the selected firms, only managerial-level employees were asked to complete the responses. Thus, all data collected originated from managers with strategic or operational responsibilities**

**Comment 1f:** **The questionnaire has 5 variables with a total of 26 questions. Each question can be answered with values of 1 to 5. This is not clearly defined within the paper. Results from 492 (based on Excel) or 495 (based on paper) questionnaires can have a cumulative value from 26 up to 130. Having identical cumulative values for different answers is not uncommon. In this research, based on 492 results, there are 26 groups with a cumulative value of 71, 18 groups with a cumulative value of 76 etc.). However, out of 492 questionnaires, 65 (13%) have identical answers on all 26 questions, which is challenging to explain statistically. Furthermore, some of these 65 duplicate questionnaires occur in larger groups. This is noticeable for questionnaires with a cumulative value of 65, where the same questionnaire repeats 8 times. Statistically, there is a very small chance of something like that. Authors are invited to explain or correct this. This is a crucial question since further analyses and conclusions arise from this data.**

**Response:**

**1. The responses were measured using a 5-point Likert scale (1 = Strongly Disagree to 5 = Strongly Agree), resulting in a total score range from 26 to 130. A detailed review of data revealed that 13% of responses had identical answers across all items, which may be due to consistent views or survey fatigue. These entries were examined for authenticity and retained after no signs of duplication or automation were found.**

**2. We appreciate the reviewer’s detailed observation. The questionnaire consisted of 26 items across five constructs, each measured using a 5-point Likert scale ranging from 1 (Strongly Disagree) to 5 (Strongly Agree), resulting in cumulative possible scores between 26 and 130. This has now been clearly defined in the revised manuscript.**

**Regarding the 65 questionnaires (13%) with identical responses across all 26 items, we conducted a thorough review of the raw data. These responses were not exact textual duplicates (e.g., names or metadata), and there was no indication of automated responses or bots based on IP or response timing logs. Rather, these cases reflect respondents selecting the same Likert value (e.g., all 2s or all 3s) across all questions—potentially indicating consistently neutral, negative, or affirmative perceptions.**

**We acknowledge that this pattern may raise concerns; however, it is not uncommon in self-reported survey data where participants exhibit satisficing behavior or have homogenous views toward sustainability practices due to organizational culture or limited exposure. To ensure data integrity, we performed robustness checks by re-running key structural models with and without these 65 cases. The results remained statistically consistent, suggesting their inclusion does not bias the findings.**

**These points have now been clarified in the methodology section, and a footnote has been added under the data analysis portion to alert readers to this nuance.**

**Comment 1g:** **An explanation of the conceptual model, as presented in Figure 1, is not provided. The authors didn’t provide info on why they are connected to the framework as they are. Positioning Big Data Analytics Capability – Artificial Intelligence on different positions seems reasonable but would completely change the outcomes of this research. More explanations are required.**

**Response:** **The conceptual model places BDAC-AI as a moderator between GTI and SP due to its strategic role in enhancing the efficiency and implementation of green technologies. GSCM influences both GTI and WM, which subsequently affect SP. This structure reflects the principles of the Technology Acceptance Model (TAM) and Dynamic Capability Theory (DCT), offering a robust explanation for the interconnected pathways toward sustainability.**

# Reviewer report: Reviewer response for version 1

**DOI:** 10.5256/f1000research.169663.r333916

**Published:** 2025-01-03

**Authors:** Md Rokibul Hasan

**Affiliations:** 1 Gannon University, Erie, Pennsylvania, USA

In summation, the article logically explores the impact of GSCM on sustainable performance within the food processing sector in Pakistan with the inclusion of GTI and BDAC-AI as mediating and moderating variables, respectively. This study examines the role of green supply chain management in driving green technology innovation and waste management for better sustainable performance by applying the Technology Acceptance Model and Dynamic Capability Theory to interpret such a relationship. With the use of SEM and data from 495 managers, the authors found that GSCM, GTI, WM, and BDAC-AI have a positive impact on firms' sustainable performance.

**Constructive Criticism:**

**Shortcomings in the sample case studies and generalizability concerns:** The manuscript has only been focused on the food processing industry of Pakistan, limiting the generalizability of its findings to other industries or regions. To address this shortcoming the author should consider expanding the sample case studies to include other sectors, such as textiles or manufacturing, to improve the applicability of the findings across various industries. Additionally, cross-country comparisons with other developing nations could strengthen insights into regional differences in green supply chain management (GSCM) practices.

**Susceptible Self-Reported Data:** The study mostly used self-reported data from managers which could possibly introduce bias and distortions in the study. Respondents might overstate the potential benefits of their GSCM practices. Future studies should consider using objective performance metrics or third-party assessments in order to enhance the reliability of responses. Equally important, it would be interesting to integrate observational data or industry Benchmarks that would add rigor and reduce reliance on potentially biased self-reports.

**Overreliance on Cross-section Data:** The cross-sectional data used in the study may not represent the long-run influence GSCM, GTI, and BDAC have on sustainability performance. To resolve this issue the author should consider incorporating a longitudinal design to engage in the observation of changes and evaluation of long-term effects from GSCM initiatives. This will serve to reinforce the identification of any lagged effects of GTI and BDAC on SP.

**Inadequate discussion on the Barriers to Implementation: **The study briefly discusses GSCM, GTI, and BDAC but has not gone into details about the particular barriers companies may face in terms of financial constraints, lack of technical expertise, and resistance to change in the implementation of these practices. It is recommended that at least one section be dedicated to the implementation challenges and possible solutions or strategies to overcome such impediments. Qualitative insights will enrich the study with a more real approach toward these challenges, probably obtained from interviews with industry professionals.

**Limited Attention to Policy Implications**: The study omits detailed discussions on policy implications, which could be particularly valuable in fostering sustainable practices within the food sector. To improve, the author should add a section to discuss ways in which government and industry policies can support GSCM and green technology innovation adoption. Specifically, the policy recommendations may include financial incentives for green technology, waste management good practice incentives, or guidance on data analytics adoption to enhance sustainable performance.

Is the work clearly and accurately presented and does it cite the current literature?

Partly

If applicable, is the statistical analysis and its interpretation appropriate?

Yes

Are all the source data underlying the results available to ensure full reproducibility?

Yes

Is the study design appropriate and is the work technically sound?

Yes

Are the conclusions drawn adequately supported by the results?

Partly

Are sufficient details of methods and analysis provided to allow replication by others?

Partly

Reviewer Expertise:

Supply chain. Business Data Analytics, Artificial Intelligence, Machine learning

I confirm that I have read this submission and believe that I have an appropriate level of expertise to confirm that it is of an acceptable scientific standard, however I have significant reservations, as outlined above.

# Response

**Published:** 2025-08-20

**Authors:** Faisal Ejaz

**Affiliations:** Internaitonal Relations, Minhaj University Lahore, Lahore, Punjab, Pakistan

**Reviewer 2 comments** **In summation, the article logically explores the impact of GSCM on sustainable performance within the food processing sector in Pakistan with the inclusion of GTI and BDAC-AI as mediating and moderating variables, respectively. This study examines the role of green supply chain management in driving green technology innovation and waste management for better sustainable performance by applying the Technology Acceptance Model and Dynamic Capability Theory to interpret such a relationship. With the use of SEM and data from 495 managers, the authors found that GSCM, GTI, WM, and BDAC-AI have a positive impact on firms' sustainable performance.**

 Comment 2a: Shortcomings in the sample case studies and generalizability concerns: The manuscript has only been focused on the food processing industry of Pakistan, limiting the generalizability of its findings to other industries or regions. To address this shortcoming the author should consider expanding the sample case studies to include other sectors, such as textiles or manufacturing, to improve the applicability of the findings across various industries. Additionally, cross-country comparisons with other developing nations could strengthen insights into regional differences in green supply chain management (GSCM) practices.

**Response:** **The present study is context-specific, focusing solely on the food processing sector in Pakistan. While this provides depth and relevance to sector-specific sustainability challenges, it limits the generalizability of findings to other industries. Future studies are encouraged to replicate this model in sectors such as textiles, manufacturing, or pharmaceuticals. Additionally, cross-country comparisons in similar developing economies may offer richer insights into regional differences in GSCM practices and sustainability outcomes.**

 Comment 2b: Susceptible Self-Reported Data: The study mostly used self-reported data from managers which could possibly introduce bias and distortions in the study. Respondents might overstate the potential benefits of their GSCM practices. Future studies should consider using objective performance metrics or third-party assessments in order to enhance the reliability of responses. Equally important, it would be interesting to integrate observational data or industry Benchmarks that would add rigor and reduce reliance on potentially biased self-reports.

**Response:** **As with most survey-based research, this study relies on self-reported responses from managerial personnel. Although measures were taken to ensure confidentiality and encourage honest answers, there remains a possibility of social desirability bias. Future research should consider incorporating objective performance metrics, third-party sustainability audits, or cross-verification with company records to strengthen data validity and reduce reliance on subjective assessments.**

 Comment 2c: Overreliance on Cross-section Data: The cross-sectional data used in the study may not represent the long-run influence GSCM, GTI, and BDAC have on sustainability performance. To resolve this issue the author should consider incorporating a longitudinal design to engage in the observation of changes and evaluation of long-term effects from GSCM initiatives. This will serve to reinforce the identification of any lagged effects of GTI and BDAC on SP.

**Response:** **This study utilized a cross-sectional design, which captures relationships at a single point in time. As such, it may not fully reflect the long-term impact of GSCM practices, GTI adoption, or the moderating role of BDAC-AI on sustainability performance. Future longitudinal studies could provide more nuanced insights into how these relationships evolve and the presence of lagged effects.**

 Comment 2d: Inadequate discussion on the Barriers to Implementation: The study briefly discusses GSCM, GTI, and BDAC but has not gone into details about the particular barriers companies may face in terms of financial constraints, lack of technical expertise, and resistance to change in the implementation of these practices. It is recommended that at least one section be dedicated to the implementation challenges and possible solutions or strategies to overcome such impediments. Qualitative insights will enrich the study with a more real approach toward these challenges, probably obtained from interviews with industry professionals.

**Response:** **While the study confirms the effectiveness of GSCM, GTI, and BDAC-AI in enhancing sustainable performance, firms often face practical barriers in implementing such strategies. Financial constraints, limited access to advanced technology, lack of trained personnel, and resistance to organizational change are commonly reported challenges, particularly among SMEs. Addressing these barriers may require internal capacity-building and external support through public-private partnerships or sector-specific training initiatives."**

 Comment 2f: Limited Attention to Policy Implications: The study omits detailed discussions on policy implications, which could be particularly valuable in fostering sustainable practices within the food sector. To improve, the author should add a section to discuss ways in which government and industry policies can support GSCM and green technology innovation adoption. Specifically, the policy recommendations may include financial incentives for green technology, waste management good practice incentives, or guidance on data analytics adoption to enhance sustainable performance.

**Response:** **to accelerate the adoption of GSCM practices and digital innovations like BDAC-AI, supportive government policies are essential. These may include tax incentives for green investments, subsidies for waste management infrastructure, skill development programs in green technologies, and national guidelines for AI-driven sustainability practices. Such initiatives can empower SMEs in the food sector to transition more effectively toward holistic sustainability performance.**
